# Supplementary material for: High-mobility, trap-free charge transport in conjugated polymer diodes
Source: Nat Commun. 2019 May 9;10:2122. doi: 10.1038/s41467-019-10188-y (PMC6509340; doi:10.1038/s41467-019-10188-y)
Supplement: Supplementary file 1 — Supplementary Information [file 41467_2019_10188_MOESM1_ESM.pdf]

Supplementary Information

High-mobility, trap-free charge transport in conjugated polymer diodes

Mark Nikolka[1*], Katharina Broch[1], John Armitage[1], David Hanifi[2], Peer J. Nowack[3], Deepak Venkateshvaran[1], Aditya Sadhanala[1], Jan Saska[4], Mark Mascal[4], Seok-Heon Jung[5], Jin-Kyun Lee[5], Iain McCulloch[6], Alberto Salleo[2], Henning Sirringhaus[1*]

[1]Optoelectronics Group, Cavendish Laboratory, JJ Thomson Avenue, Cambridge CB3 0HE, United Kingdom.

[2]Department of Material Science and Engineering, Stanford University, Stanford, CA 94305, United States

[3]Faculty of Natural Sciences, Imperial College London, Exhibition Road, SW7 2AZ, United Kingdom

[4]Department of Chemistry, University of California, Davis, CA 95616, United States

[5]Department of Polymer Science & Engineering, Inha University, Incheon 402-751, South Korea

[6]King Abdullah University of Science and Technology (KAUST), Kaust Solar Center (KSC), Thuwal 23955-6900, Saudi Arabia and Department of Chemistry and Centre for Plastic Imperial College London, Exhibition Road, SW7 2AZ, United Kingdom

Supplementary Note 1 – SCLC characteristics in devices without traps or discrete trap levels

The concept of space-charge limited currents arises in device geometries where charge carriers are injected from an electrode and, at sufficiently high charge density, the associated space charge modifies the electrostatic potential in the device from that induced by the external, applied electric field. This cloud arises from the incapacity of a material to carry away the emitted charges fast enough. Therefore, space charge generally only occurs in a dielectric media since only here charges cannot be screened or neutralized fast enough. The current density at which a space charge is formed is hence a direct measure of a material's charge carrier mobility. In a planar diode geometry and in the absence of trap states in the material, the mobility is related to the current density via Mott-Gurney's or Child's law:

$$J = \frac{9\varepsilon\varepsilon_0\mu V^2}{8L^3} \quad , \tag{1}$$

where J is the current density, ε is the material's dielectric constant (~3.5 for most organic semiconductors), ε_0 is the permittivity of free space, V is the applied voltage and L is the thickness of the dielectric material.

In the presence of trap states confined in a single or multiple discrete energy levels, the J-V characteristics will only follow Child's law for high applied voltages. When plotted on a ln-ln (or log-log) plot the J-V characteristics will ideally exhibit 4 characteristic regimes: (i) an Ohmic regime in which the material is behaving like a resistor showing $J \propto V^m$ with a slope of $m = 1$; (ii) an SCLC regime in which Child's law is obeyed, i.e. $m = 2$, but the extracted mobility is low and limited by the presence of trap states; (iii) a trap filling regime in which $m >> 2$; (iv) The trap-free SCLC regime with $m = 2$, in which all the trap states are filled and in which the extracted mobility is no longer affected by traps and is much higher than in regime 2. There may be modifications of the extracted voltage dependence, for example if the mobility is dependent on the magnitude of the applied electric field.

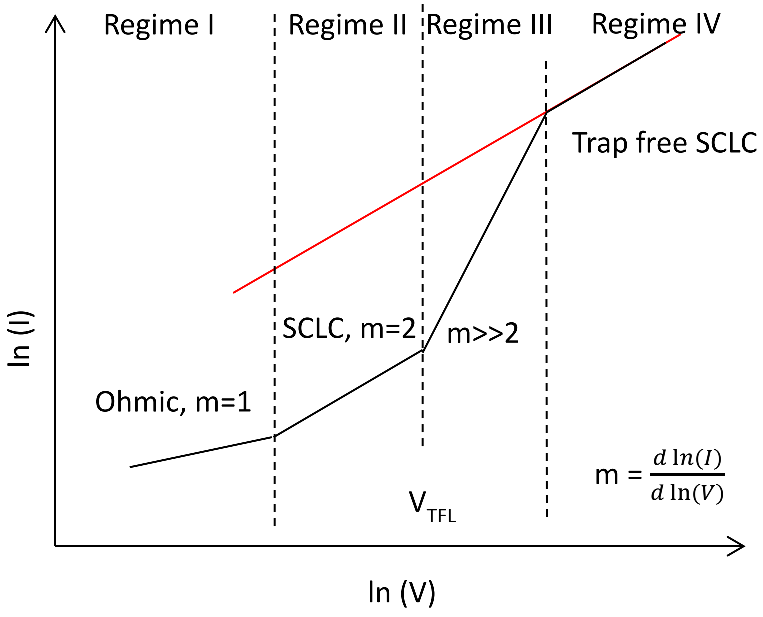

Supplementary Figure 1: Sketch of an idealized SCLC-diode characteristic. *Ideal SCLC-diode characteristics on a ln-ln (log-log) scale. The 4 regions that are clearly identifiable are (I) Ohmic regime (II) trap-limited SCLC (III) trap filling and (IV) trap-free SCLC.*

Supplementary Note 2 – Degradation, reproducibility and effects of heating

For the investigation of temperature dependent SCLC measurements, it is crucial that device stability is sufficient to draw reliable conclusions. We find that our DPP-BTz diodes show very good stability over the course of the measurement (5h). Within the voltage range applied the *J-V* characteristics is also not affected by hysteresis or polymer degradation effects at the high current densities present. Although the on-current slightly reduces (we attribute this to a slow evaporation of solvent), the overall shape of the device characteristics remains highly stable even after 46 repetitive measurements.

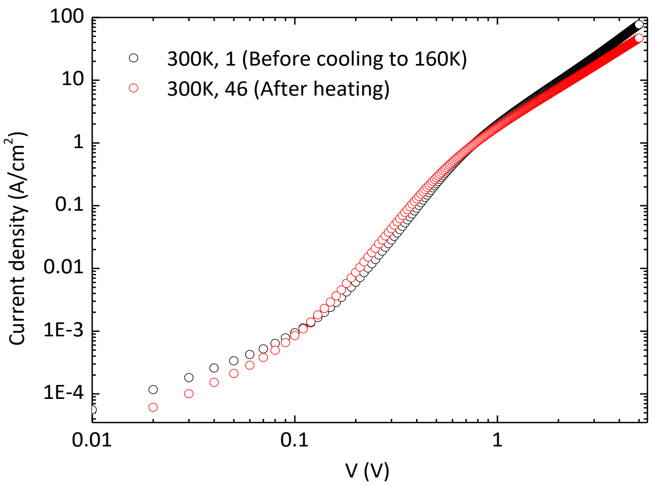

Supplementary Figure 2: Stability of diodes during measurement. *Stability of DPP-BTz SCLC diodes over the course of a full temperature cycle cooling from 300K to 160K and subsequently heating to 300K. The black line represents the initial SCLC characteristics whereas the red line represents the SCLC-characteristics after cooling and heating including 46 measurement sweeps from 1 to 5V.*

We have also investigated the effect of heating on our SCLC diodes to exclude any measurement artefact. As precaution, we measured the *J-V* characteristics 3 times to monitor if degradation is taking place between consecutive sweeps. We find that even at temperatures as low as 180K, measurements are highly reproducible as evident by the identical $m = dLn(J)/dLn(V)$ plots in Supplementary Figure 3. Nevertheless, once applied voltages are increased beyond 5V, we do see that the plots of *m* exhibit an inflation point followed by a gradual reduction of the slope. We attribute this behavior to significant heating caused by an increase of the power dissipation from 140 W/cm^2 at 200 K to 830 W/cm^2 at 180 K after the measurement range is extended from 5 V to 7 V. The resulting degradation is permanent and any consecutive measurement will follow the degraded characteristics; only a further extension of the measurement range will lead to an additional loss of current. For all data presented in this work, we have made sure that the measurement range was chosen in such a way that characteristics did not get close to the inflection point and therefore, we avoided the onset of this heat induced degradation.

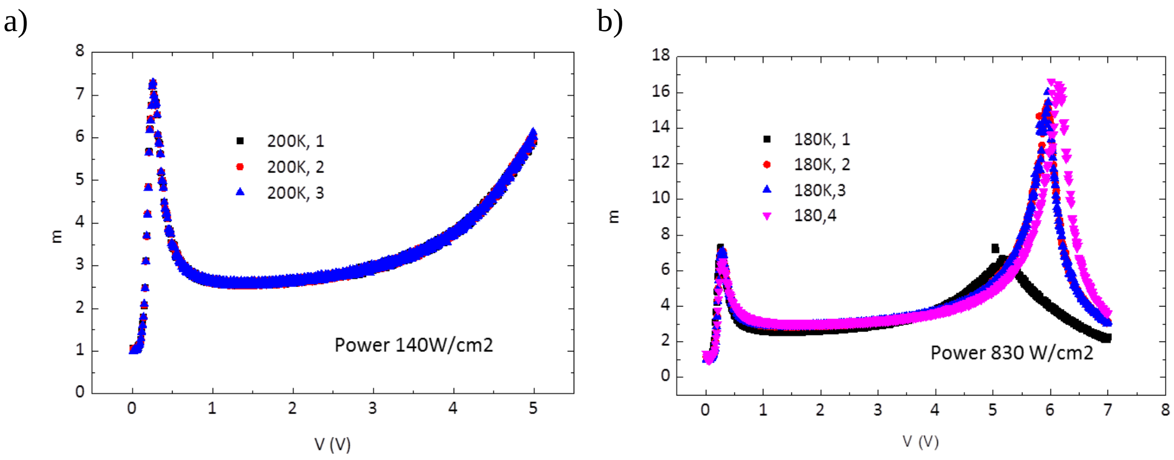

Supplementary Figure 3: Heat stability of DPP-BTZ diodes. *Reproducibility of DPP-BTz SCLC diode characteristics; a) Reproducibility of the m = dln(J)/dln(V) slope for a DPP-BTz SCLC diode measured three times at a temperature of 200K; b) plot of m after the measurement range is increased from 5 to 7 V.*

All the SCLC diodes we present in this work were fabricated on glass substrates. We have also prepared devices on silicon substrates to exclude that the lower thermal conductivity of glass did not lead to an excessive heating and thus to measurement artefacts. However, we find that the choice of substrate did not have a major impact on the SCLC characteristics of our diodes (Supplementary Figure 4).

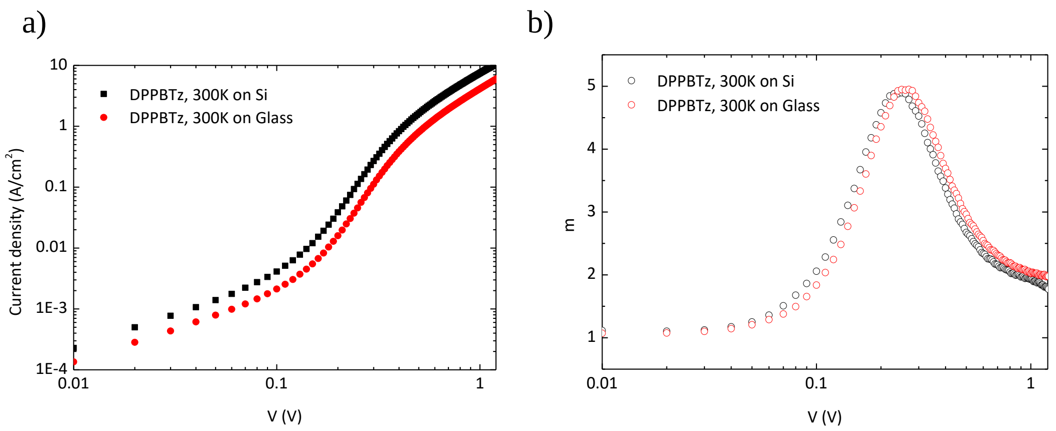

Supplementary Figure 4: Impact of substrate on diode characteristics. *a) Comparison of DPP-BTz SCLC diode characteristics fabricated on silicon (black) and on glass (red); b) corresponding derivative m of the I-V characteristics.*

Supplementary Note 3 – TD-SCLC measurements

We have used the TD-SCLC method to extract the density of trap states from our diode characteristics. The theoretical details and derivation of TD-SCLC can be found elsewhere[1,2,3] and here, we only give a summary of the key formulas to facilitate readability of the paper as well as a key amendment we have added to the deconvolution mechanism used in this method.

The shape of the *J-V* characteristics is directly related to the increment of space charge with respect to the shift of the Fermi energy *dn/dE* and related by

$$\frac{dn}{dE} = \frac{V}{k_B T}\frac{\varepsilon\varepsilon_0}{eL^2}\frac{(2m-1)}{m^2}(1+C) \quad , \tag{2}$$

with

$$\mathrm{C} = \frac{B(2m-1)+B^2(3m-2)+d[\ln(1+B)]/dlnV}{1+B(m-1)} \quad , \tag{3}$$

and

$$\mathrm{B} = \frac{-\left[\frac{d\,m}{d\,ln\,V}\right]}{m(m-1)(2m-1)} \quad . \tag{4}$$

Where *m=m(V,T)* is the slope of the *J-V* characteristics on a ln-ln (or alternatively log-log) scale (*m = dln J/dln V*), k_B is the Boltzmann constant, *L* is the thickness of the diode, *e* is the elementary charge and ε the dielectric constant of the semiconductor (assumed to be 3.5).

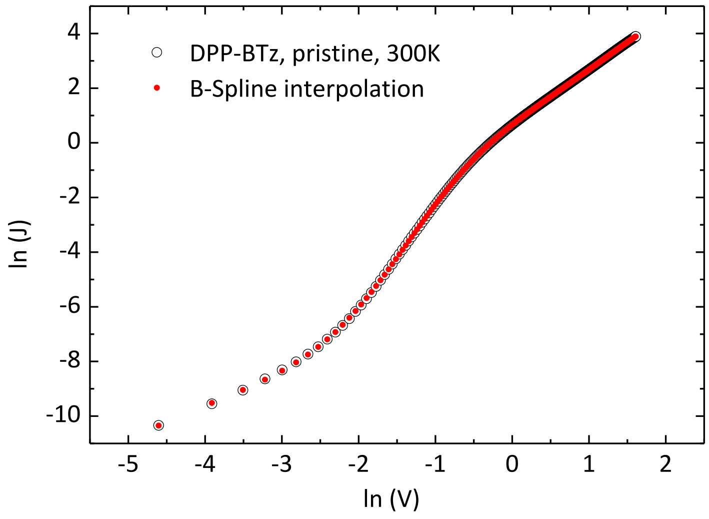

Supplementary Figure 5: Smoothing of raw data for TD-SCLC analysis. *Raw data (black) and smoothing B-spline (smoothing factor of 0.01) fitted to a pristine DPP-BTz SCLC diode.*

In agreement with what has been observed elsewhere [*Ref. 17*], we found that running the above analysis from the raw *J-V* data introduces a substantial amount of noise – especially due to the $d[ln(1+B)]/dlnV$ term, which involves a third derivative of ln J with respect to ln V and is thus extremely sensitive to small amounts of measurement noise in the raw data. This resulted in an unphysical scatter of the *dn/dE* values. Therefore, we instead, fitted smoothing Bayesian-splines (henceforth referred to as B-splines) with a fixed error of 1% to all our raw data (Supplementary Figure 5). Even though by naked eye these fits were indistinguishable from the raw data, they enabled a clean differentiation of the data down to the 3rd order and resulted in reliable extraction of *dn/dE* values.

The activation energy was extracted from the *J-V* characteristics for 50 evenly spread data points. A smoothing B-spline was subsequently applied to the extracted data using the same conditions as used for the *J-V* raw data. The activation energy was then used to translate the voltage scale of the extracted *dn/dE(V)* values into an energy scale (i.e. the position of the Fermi level). In order to do so, it has to be corrected by the dominant energy E_D:

$$E_D = E_A + \frac{(3-4m)n\prime}{(2m-1)(m-1)m} K_B T \quad , \tag{5}$$

with n' being the experimentally determined derivative of the activation energy with respect to applied voltage [$n' = -d(E_A/k_BT))/d(\ln V)$].

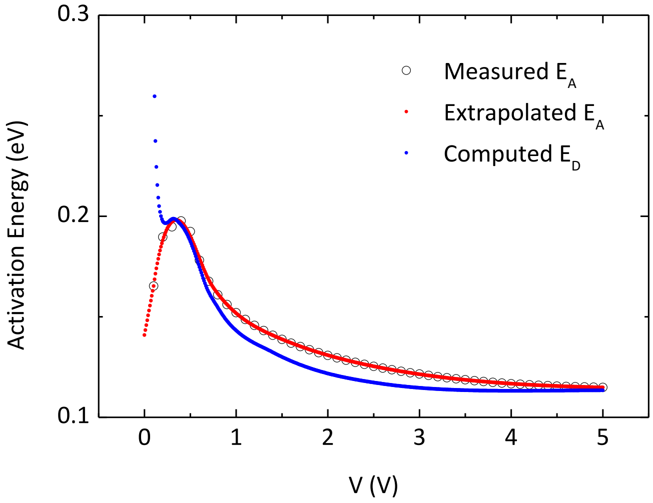

Supplementary Figure 6: Activation Energy extracted for DPP-BTz SCLC diodes. *Extracted activation energy as well as computed* E_D *for a pristine DPP-BTz SCLC diode. The reduction of* E_A *for voltages V<0.5 V is an artefact of noise affecting the measurements at low temperatures and low voltages. Since data for V < 0.5 V was not used for our analysis, this artefact did not have an impact on the extracted dn/dE(E) values.*

From the *dn/dE(E)* values, the density of states can be extracted. The two quantities are related by the Fermi-Dirac distribution according to:

$$\frac{dn}{dE} = \int_E \mathrm{h(E)} \frac{d\, f(E-E_F)}{d(E-E_F)} d\mathbf{S} \quad , \tag{6}$$

where *h(E)* is the density of states and $f(E-E_F)$ is the Fermi-Dirac function. In the literature *h(E)* and *f(E)* have previously been deconvoluted using cubic splines. However, we found that in our case this method introduces a significant scatter in the coefficients. We have therefore decided to compare only the more reliable *dn/dE* values in the main text.

We have however applied a deconvolution method based on 3rd order ridge regressions giving more reliable fits and continuous coefficients[4]. The data set was regularized to a 1000-point grid and for each point the regression's coefficients A_k, B_k, C_k and D_k (Supplementary Figure 7) were determined according to:

$$\frac{dn}{dE} = A_k E^3 + B_k E^2 + C_k E + D_k \tag{7}$$

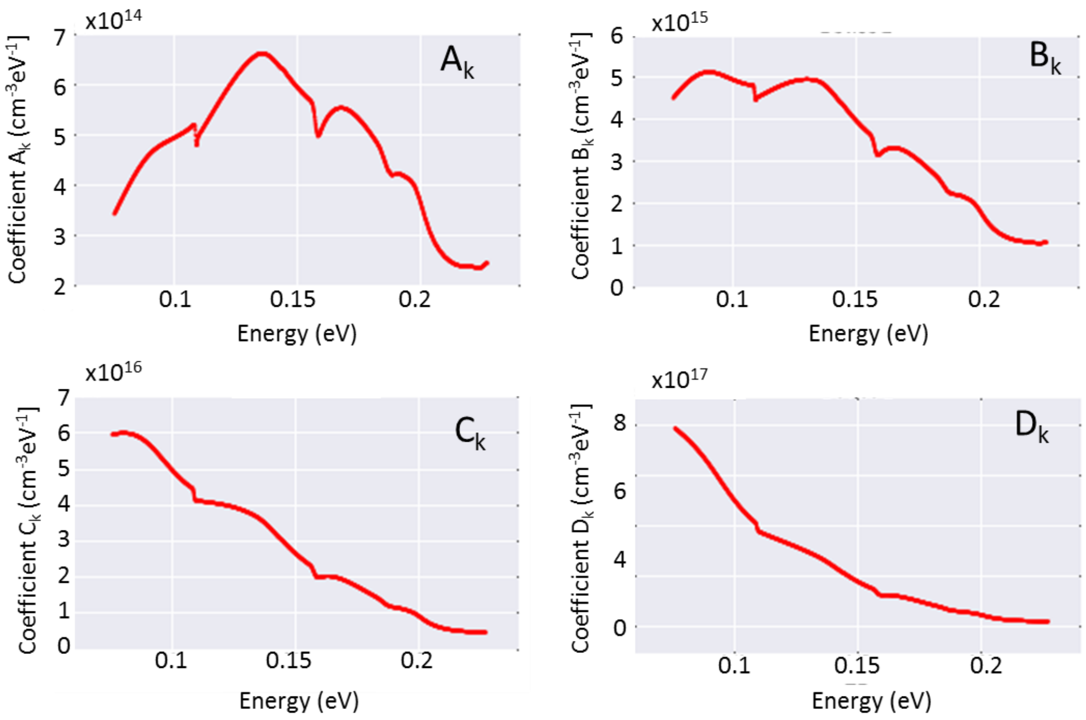

Supplementary Figure 7: Extraction of the DOS of DPP-BTz using ridge regression. *Coefficients A_k, B_k, C_k and D_k extracted from a 3rd order ridge regression to the dn/dE data for a DPP-BTz device with additive at 180K.*

The density of states (DOS) was subsequently calculated by multiplying the coefficients A_k, B_k, C_k and D_k with the corresponding moments of the Fermi-Dirac function (see *Ref.* 5 for more detail). However, we observed that the obtained DOS was almost indistinguishable from the dn/dE values reported in the paper (Supplementary Figure 8).

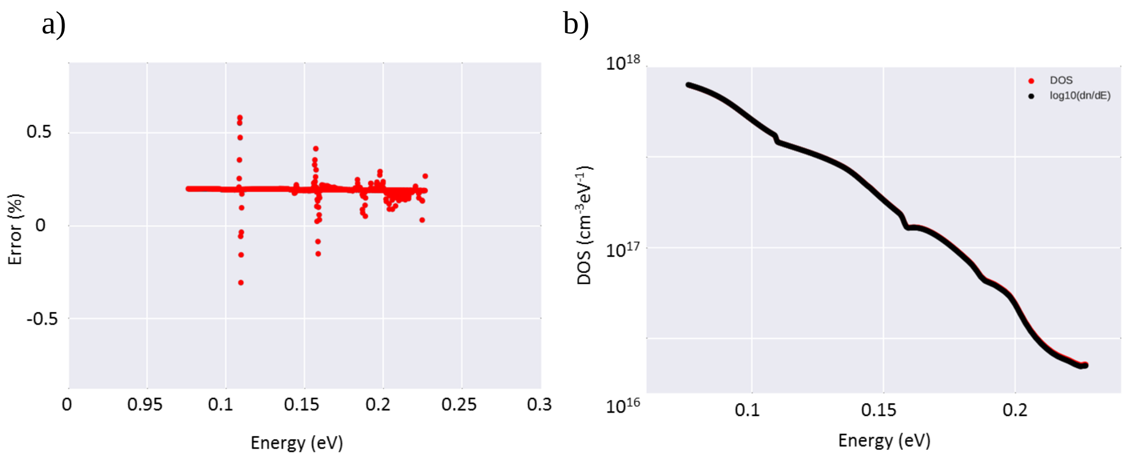

Supplementary Figure 8: Comparison of DOS and dn/dE analysis. *a) Fitting error of a 3rd order ridge regression to the dn/dE data; b) comparison of dn/dE values (black line) and convoluted DOS (red line) using coefficients A_k, B_k, C_k and D_k of the ridge regression as well as the corresponding moments of the Fermi-Dirac distribution function at 180K.*

From the data the statistical shift can also be extracted, which gives useful information on the pinning of the Fermi level and the reliability of the reconstruction of trap densities extracted with TD-SCLC spectroscopy. The statistical shift arises from the temperature dependence of the Fermi level in the case of space-charge-free conductivity. It can be written by using the linear term of a Taylor expansion around the temperature independent value E_{F0} at $T = 0$ K: $E_F(T) = E_{F0} - \gamma_F k_B T$ with γ_F being the statistical shift of the Fermi level. The so-called Meyer-Neldel parameter G can be used to relate the statistical shift to the slopes m and n of the J-V characteristics and the activation energy, respectively, according to:

$$G = \frac{\gamma_F}{E_A} = \frac{1}{K_B T}\left(1 - \frac{m-1}{n}\right) \tag{8}$$

For the polymer DPP-BTz, we show the statistical shift/Meyer-Neldel parameter explicitly (Supplementary Figure 9). In all our polymer devices we observe a negative Meyer-Neldel parameter for high energies which correlates to a Fermi-level pinning at trap states. For a Meyer-Neldel parameter below approx. 30 eV^{-1}, the reconstruction of the density of trap states yields reliable results. A strong shift of the Meyer-Neldel parameter towards high negative values at lower energies, on the other hand, suggests that the Fermi level jumps significantly between two sets of states (e.g. one delocalized and one localized). In this region, the reconstruction of the *dn/dE* values cannot be trusted and hence, such data points have been removed for all materials shown in this work.

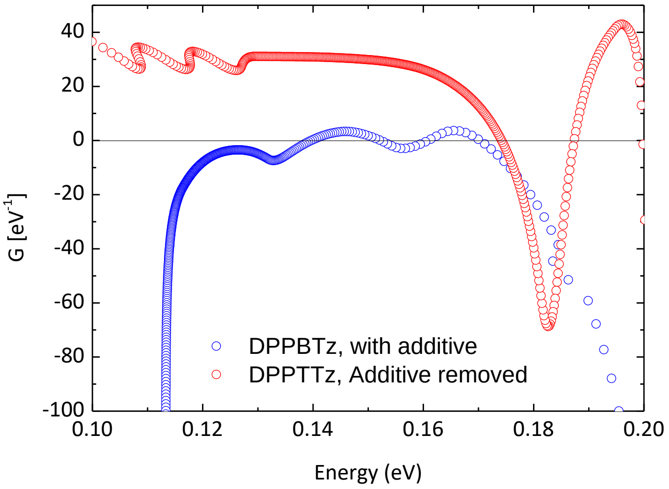

Supplementary Figure 9: Reliability of DOS reconstruction. *Meyer-Neldel parameter of a DPP-BTz SCLC diode with and without an additive.*

For the determination of SCLC mobilities, a precise knowledge of film thickness is essential. For all devices for which we report SCLC mobilities in this work, we have measured the film thickness using atomic force microscopy (AFM). The film thickness was measured by removing the polymer film with a scalpel and measuring the resulting trench both on gold (bottom electrode) as well as on the glass substrate.

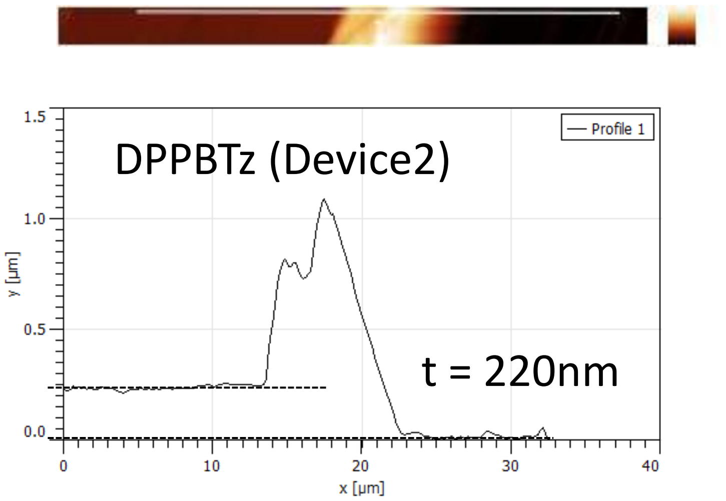

Supplementary Figure 10: Film thickness of DPP-BTz diodes using AFM. *Measured film thicknesses of DPPBTz diodes using atomic force microscopy (AFM). The film thickness was extracted between the dashed horizontal lines.*

Supplementary Note 4 – Low temperature measurements for other low disorder polymers

<u>DPP-BTz without additive:</u> After the solvent additive is removed, the current density is much reduced, but it is still possible to measure the temperature dependence of the *J-V* characteristics. There is no more maximum in the slope *m* between 0 and 1 V which is indicative of the fast filling of traps followed by an extended plateau with constant slope at higher voltages. Instead, the slope *m* is increasing monotonically until a plateau is reached at much higher values of *m* (corresponding to values of *r* between 3 and 6)

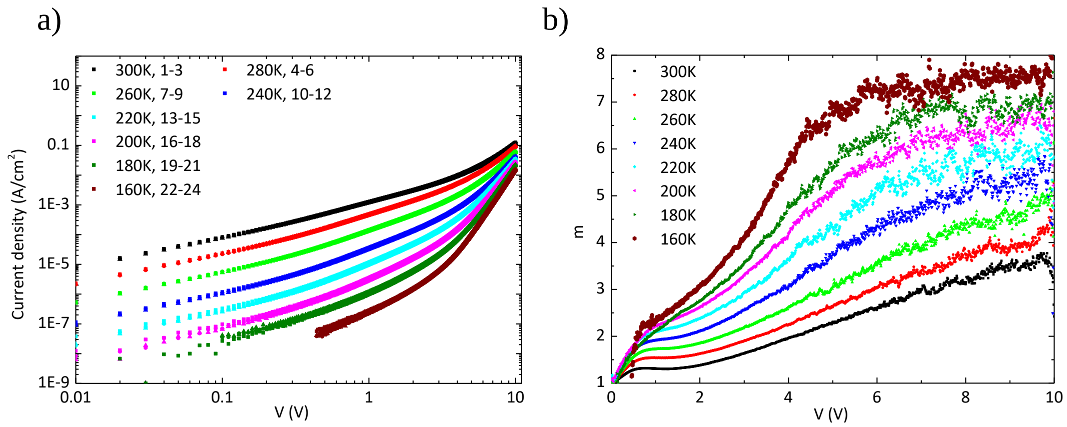

Supplementary Figure 11: Low-T measurements on DPP-BTz diodes without additive. *a) Log-log J-V characteristics of a DPP-BTz diode after the solvent additive has been removed, measured at temperatures between 300K and 160K; each characteristic was recorded 3 times to exclude degradation; b) Corresponding plot of m; the width of the trap distribution cannot be extracted without a large error from the analysis of the r values because of their pronounced voltage dependence, but values of r are significantly larger than* k_BT.

<u>DPP-DTT with additive:</u>

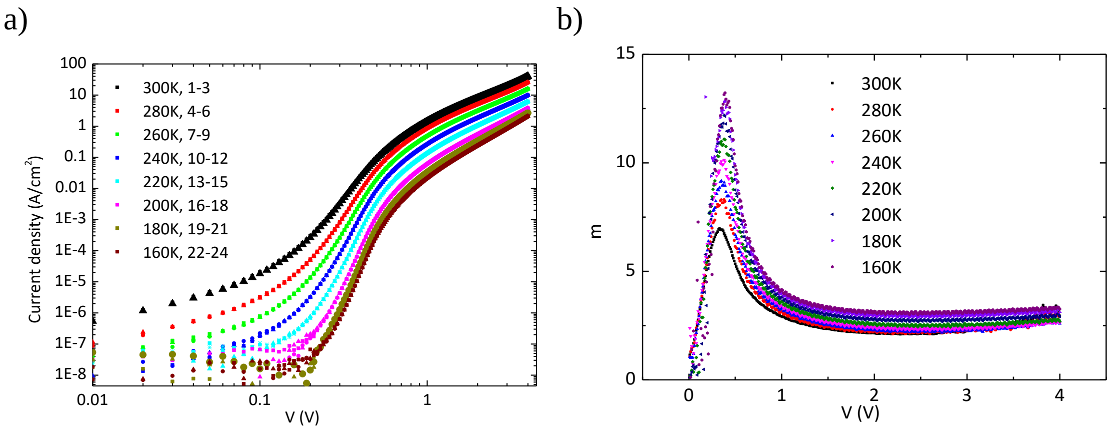

Supplementary Figure 12: Low-T measurements on DPP-DTT diodes with additive. *a) Log-log J-V characteristics of a DPP-DTT diode with solvent additive measured at temperatures between 300K and 160K; each characteristic was recorded 3 times to exclude degradation; b) Corresponding plot of m; the width of the trap distribution can be extracted from the extended plateau region between 1.5 and 4 V.*

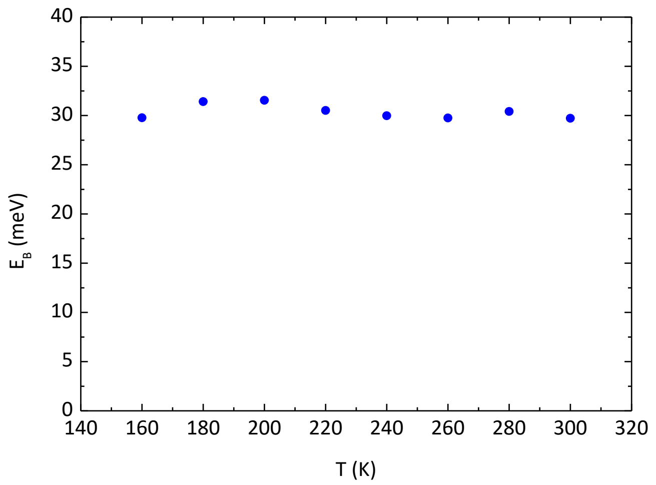

Supplementary Figure 13: Trap distribution of DPP-DTT diodes with additive. *Value of the width of the trap distribution $E_B = r \cdot k_BT$ extracted from the plateau region of the slope $m = r+1$ for a DPP-DTT diode in the temperature range from 300K to 160K.*

<u>DPP-DTT without additive:</u> We subsequently annealed our devices at 90 °C in an N_2 glove box to remove the solvent additive completely (Fig. S14). As anticipated these devices exhibit significantly lower current levels. A complete trap filling and subsequent trap-free SCLC behavior can no longer be observed.

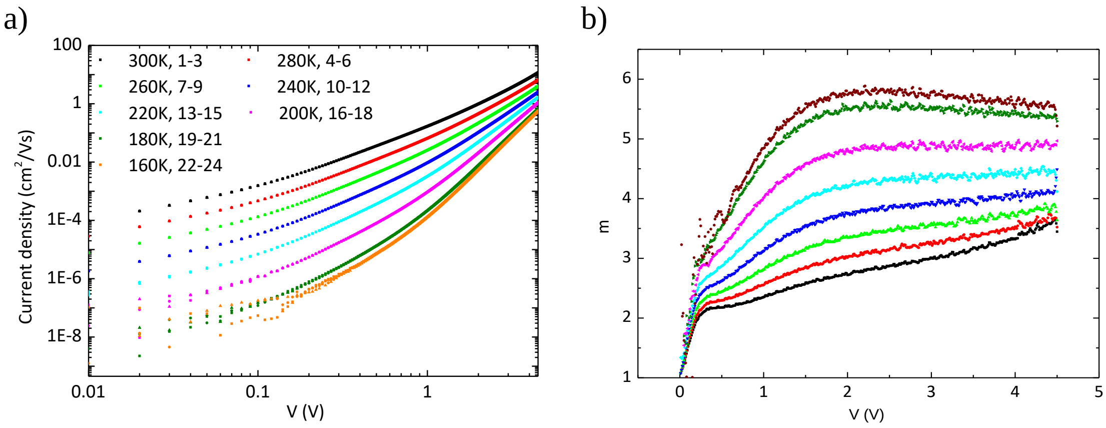

Supplementary Figure 14: Low-T measurements on DPP-DTT diodes without additives. *a) Log-log J-V characteristics of a DPP-DTT diode (same device as in Supplementary Figures 12,13) after the solvent additive has been removed, measured at temperatures between 300K and 160K; each characteristic was recorded 3 times to exclude degradation; b) Corresponding plot of m.*

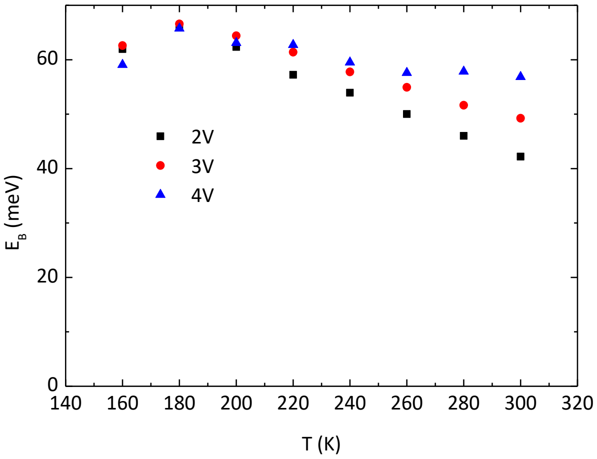

Supplementary Figure 15: Trap distribution of DPP-DTT diodes without additive. *Value of the width of the trap distribution $E_B = r \cdot k_BT$ extracted for DPP-DTT diodes after solvent removal. Since the slope m did not exhibit a clear plateau region, E_B was extracted for several voltages. At high temperatures the extracted E_B values depend on voltage but at low temperatures they converge towards a value that is significantly higher than in the presence of an additive.*

<u>IDT-BT with additive:</u> IDT-BT SCLC-diodes used for low temperature measurements were slightly thinner (110 nm) and did not show the same ideal SCLC-characteristics as DPP-BTz or DPP-DTT or the thicker IDT-BT device shown in the paper (Figure 3). Due to its amorphous microstructure the solvent additive evaporates faster in these devices, making it harder to fabricate a device of sufficient quality for TD-SCLC analysis. Furthermore, there remains a small injection barrier in all our IDT-BT devices. Especially at lower voltages we did not observe an Ohmic behavior (m = 1) and the measured currents were very small. These devices also exhibited an SCLC slope of $m < 2$ which is furthermore suggestive of injection limitations. Nevertheless, we did observe a similar plateau in the slope (m) as seen for DPP-BTz and DPP-DTT. Additionally, we did not find major differences in the crucial trap filling domain, making this device as suited for the TD-SCLC analysis as the device shown in Figure 3.

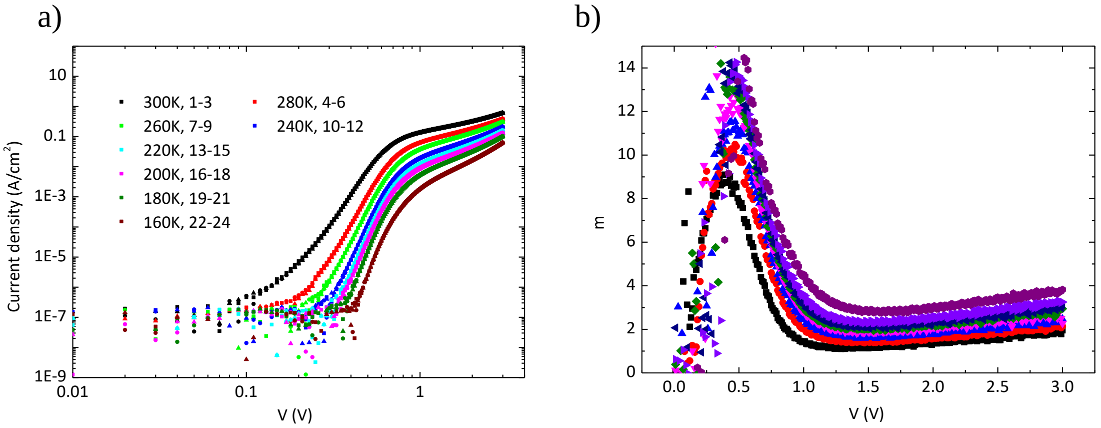

Supplementary Figure 16: Low-T measurements on IDT-BT diodes with additive. *a) Log-log J-V characteristics of an IDT-BT diode with solvent additive measured at temperatures between 300K and 160K; each characteristic was recorded 3 times to exclude degradation; b) Corresponding plot of m; the width of the trap distribution can be extracted from the extended, approximate plateau region between 1.5 and 3 V.*

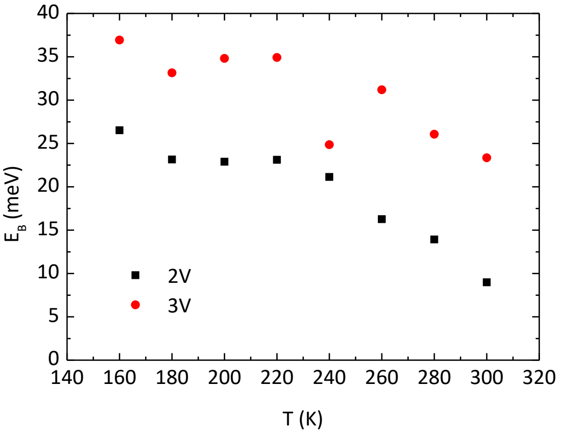

Supplementary Figure 17: Trap distribution of IDT-BT diodes with additive. *Value of the width of the trap distribution $E_B = r \cdot k_BT$ extracted for IDT-BT diodes before solvent removal. Since the slope m did not exhibit a perfect plateau, E_B was extracted for several voltages. Since diodes did not show perfect characteristics, we can only obtain values for E_B with a large uncertainty. For consistency with other results published in this work we therefore report the highest values for E_B extracted at a voltage of 2V. Also, at higher temperatures the extracted r values are less than 1, again suggesting injection limitations. Hence, values of E_B obtained at lower temperature should be considered more reliable. This is also evident by the tailing-off of the extracted values at lower T.*

IDT-BT without additive:

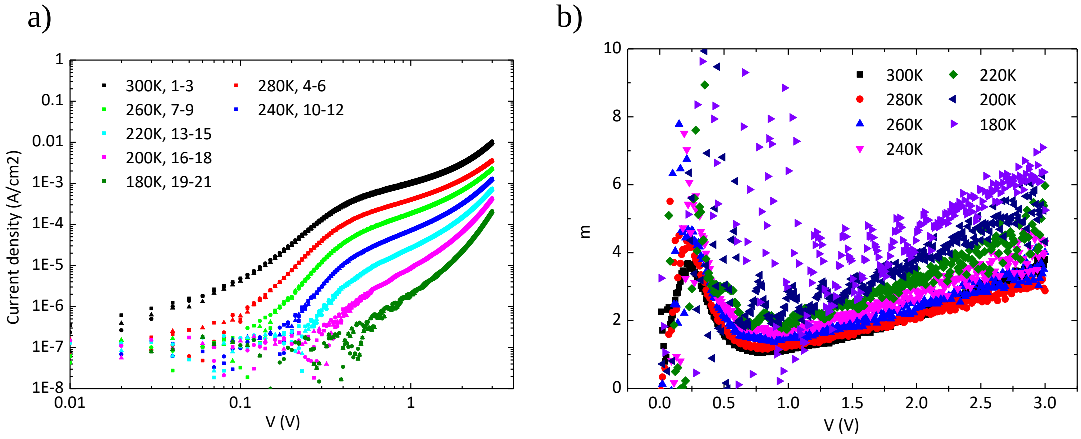

Supplementary Figure 18: Low-T measurements on IDT-BT diodes without additive. *a) Log-log J-V characteristics of a IDT-BT diode after the solvent additive has been removed measured at temperatures between 300K and 160K; each characteristic was recorded 3 times to exclude degradation; b) Corresponding plot of m; the width of the trap distribution cannot be extracted as the previous extended plateau has vanished. Due to lower current densities, recorded data exhibits increased scattering at lower temperatures.*

MEH-PPV without/with additive:

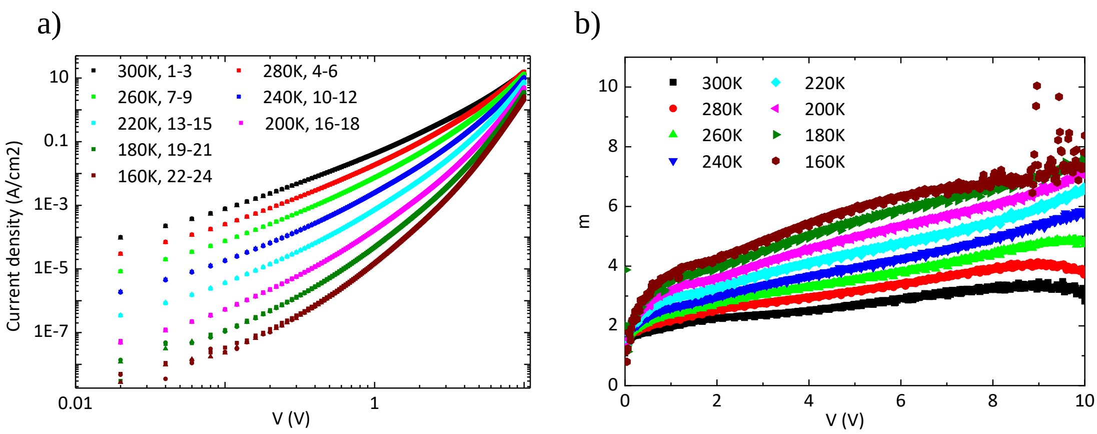

Supplementary Figure 19: Low-T measurements on MEH-PPV diodes with additive. *a) Log-log J-V characteristics of a MEH-PPV diode measured at temperatures between 300K and 160K; each characteristic was recorded 3 times to exclude degradation; b) Corresponding plot of m; the width of the trap distribution cannot be easily extracted as there is an additional field dependence*.

It is important to note that MEH-PPV devices did not perform significantly differently in the presence or absence of the solvent additive. In contrast to the low disorder polymers we have investigated, our MEH-PPV devices furthermore do not exhibit a plateau at lower voltages where the slope m is constant with voltage and hence field independent. We attribute this to the field dependence of the charge carrier mobility which we do not account for in our analysis. Nevertheless, despite the uncertainty induced by this, we cannot extract values lower than 55 meV for the width of the trap distribution, which is at least twice as high as for the low disorder polymers in the presence of an additive.

Our data on MEH-PPV are qualitatively very similar to data reported in the literature[6,7]. Clearly, for polymers such as MEH-PPV an Extended Gaussian disorder model (EGDM) as used in the literature provides a more accurate model of the data; our simple analysis based on r values on the other hand can only identify an approximated lower bound on the energetic disorder. However, it is applied here to provide a comparison with the low disorder polymers whose SCLC characteristics are limited by water-induced traps and not by inherent energetic disorder like MEH-PPV.

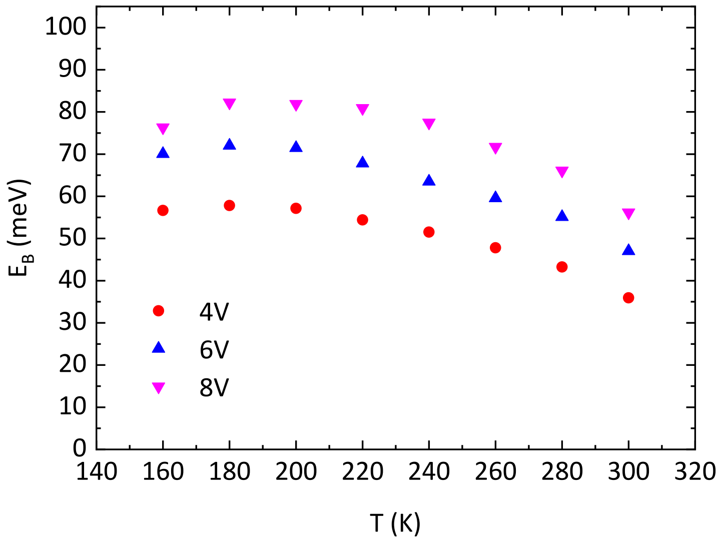

Supplementary Figure 20: Trap distribution of MEH-PPV diodes. *Value of the width of the trap distribution $E_B = r \cdot k_B T$ extracted for MEH-PPV diodes. Since the slope m did not exhibit a perfect plateau, E_B was extracted for several voltages. Extracted values for E_B are not field invariant and only temperature invariant at lower temperatures. It hence confirms that our method cannot be applied without considering an additional field dependence of the charge carrier mobility and therefore, an Extended Gaussian disorder model (EGDM) would be better suited to fit the data.*

Supplementary Note 5 – TD-SCLC measurements on MEH-PPV

We measured SCLC diodes fabricated with and without a solvent additive for 70 nm thick films of the polymer MEH-PPV (Supplementary Figure 21a). We find that in the case of MEH-PPV, annealing does not have a major influence on the diode characteristics; the presence of a solvent additive hence does not have a beneficial impact on device performance in the case of MEH-PPV diodes. This result is in stark contrast to what we observe for all of the low-disorder donor-acceptor polymers we have investigated in this paper, which show significant degradation once trap-passivating solvents are removed. We attribute this to the higher intrinsic disorder of MEH-PPV, which concomitantly exhibits similar FET and bulk mobilities; water induced traps in this material hence do not seem to be the dominant transport limiting factor.

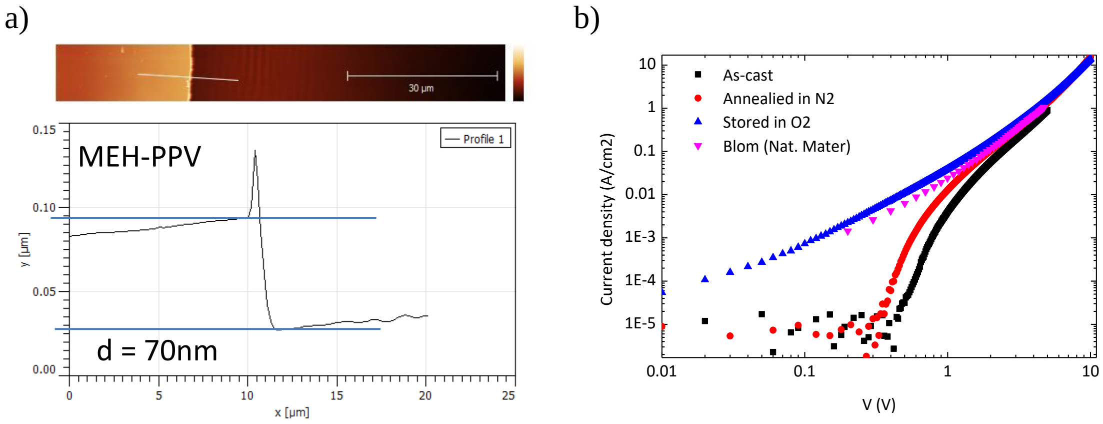

Supplementary Figure 21: Properties of MEH-PPV diodes. *a) Thickness of MEH-PPV films measured by atomic-force microscopy (AFM); b) Comparison of MEH-PPV diode characteristics processed with (black) and without (red) an additive as well as characteristics after air exposure (blue). Additionally, literature data (scaled) are plotted as a reference. The data were normalized for thickness (assuming a $1/d^3$ scaling) for easier comparison with data published elsewhere*[6].

We furthermore observed that after processing, our MEH-PPV diodes exhibited a substantial injection barrier that manifests itself as a suppression of the current at low applied voltages (< 0.3V) (red/black dots in Supplementary Figure 21b). After exposure to air, the low voltage performance of our diodes improves, whereas the performance at higher voltages remains unchanged. We associate these differences to oxygen doping which leads to better injection from the contacts and seems necessary to obtain an MEH-PPV SCLC device with ideal performance that matches data published in the literature (Supplementary Figure 21b, magenta triangles).

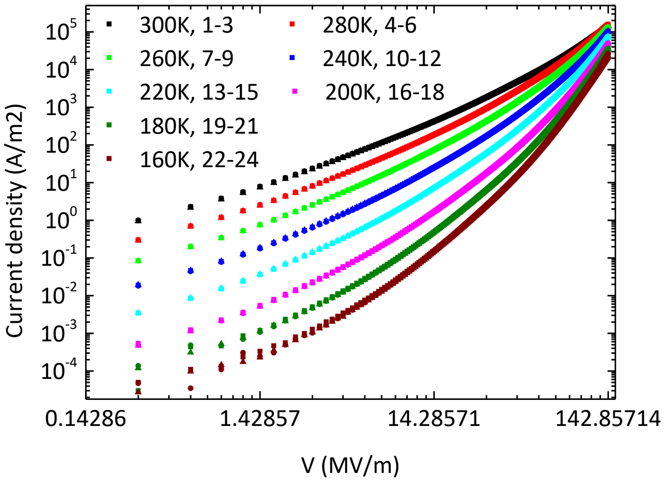

Supplementary Figure 22: Low-T measurements on MEH-PPV diodes. *a) Log-log J-V characteristics of a MEH-PPV diode measured at temperatures between 300K and 160K (same as shown in Supplementary Figure 21 but scaled for the electric field for easier comparison to literature data)*

We used the temperature dependent SCLC diode data measured for MEH-PPV to perform TD-SCLC spectroscopy. For MEH-PPV we did not observe a difference in the characteristics in the presence or absence of solvent additives. Therefore, here we only show MEH-PPV devices that have been annealed in nitrogen, which resulted in slightly improved characteristics (See Supplementary Figure 21).

a) b)

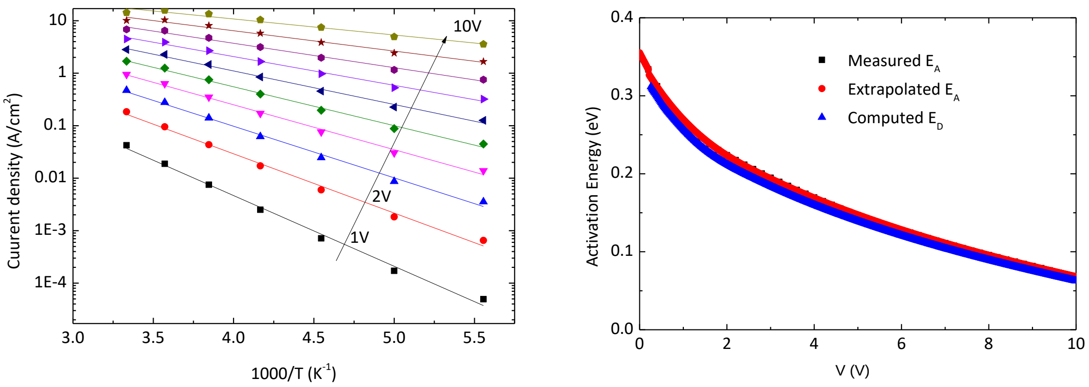

Supplementary Figure 23: Activation energy extracted from MEH-PPV diodes. *a) Arrhenius-type plot of the temperature dependence of the current density at different applied voltages for a MEH-PPV diodes; b) Corresponding extracted activation energy* E_A *as well as computed* E_D *values for MEH-PPV SCLC diodes.*

We would like to stress that since the high field performance of our MEH-PPV diodes remains unchanged by oxygen exposure, the steeper slope seen after processing may be an artefact of contact resistance (with the current rising more steeply at higher voltages when overcoming contact resistance limitations). We nevertheless extracted the *dn/dE* values for a device before oxygen exposure and did not find a substantial impact on the overall trap density (Supplementary Figure 24). Yet, due to the non-ideal SCLC characteristics of these devices (as evident as well by the large scatter in the data), we consider this data less reliable at higher energies.

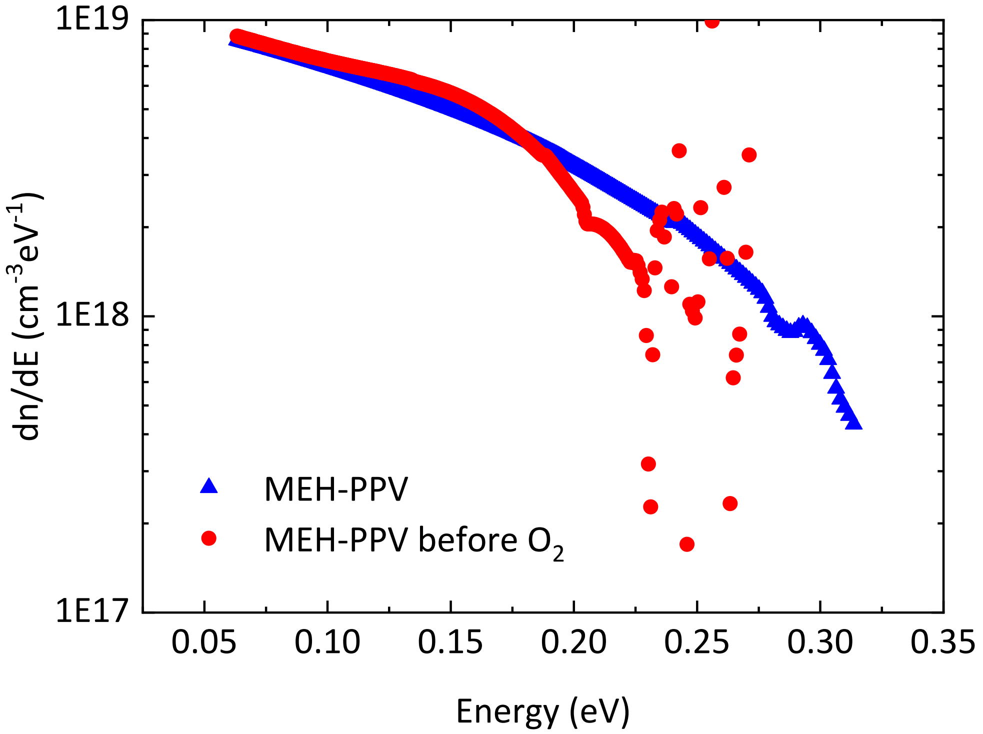

Supplementary Figure 24: Effect of air exposure on extracted dn/dE of MEH-PPV. *Comparison of the extracted dn/dE values for MEH-PPV films before (red) and after oxygen/air (blue) exposure.*

Supplementary Note 6 – TD-SCLC measurements on DPP-DTT

We measured DPP-DTT SCLC diodes fabricated with a solvent additive in the temperature range from 300K to 160K (Supplementary Note 4). The extracted activation energy as well as the computed E_D values for a pristine DPP-DTT SCLC diode are shown in Supplementary Figure 25.

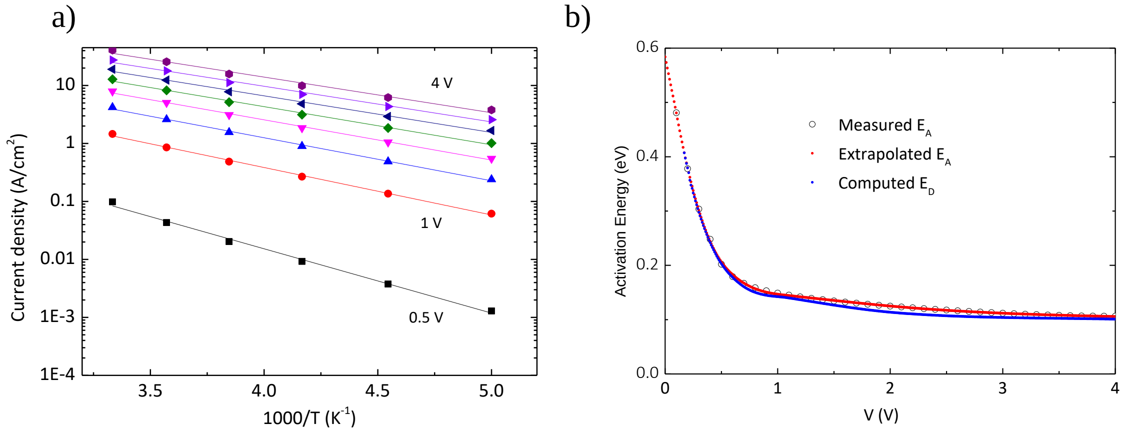

Supplementary Figure 25: Activation energy of DPP-DTT diodes with additive. *a) Arrhenius-type plot of the temperature dependence of the current density at different applied voltages for a pristine DPP-DTT diode with residual solvent additive; b) Corresponding extracted activation energy E_A as well as computed E_D values.*

We subsequently annealed our devices at 90 °C for 1h in an N_2 glove box to remove the solvent additive completely (Supplementary Figure 26). The extracted activation energy as well as computed E_D values for a DPP-DTT SCLC diode with the additive removed are shown in Supplementary Figure 26.

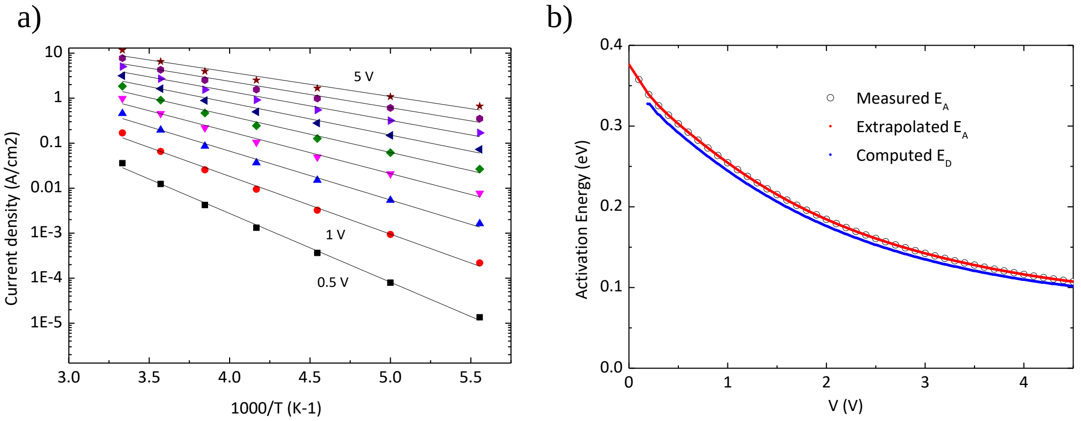

Supplementary Figure 26: Activation energy of DPP-DTT diodes without additive. *a) Arrhenius-type plot of the temperature dependence of the current density at different applied voltages for a DPP-DTT diode with the solvent additive removed; b) Corresponding extracted activation energy E_A as well as computed E_D values.*

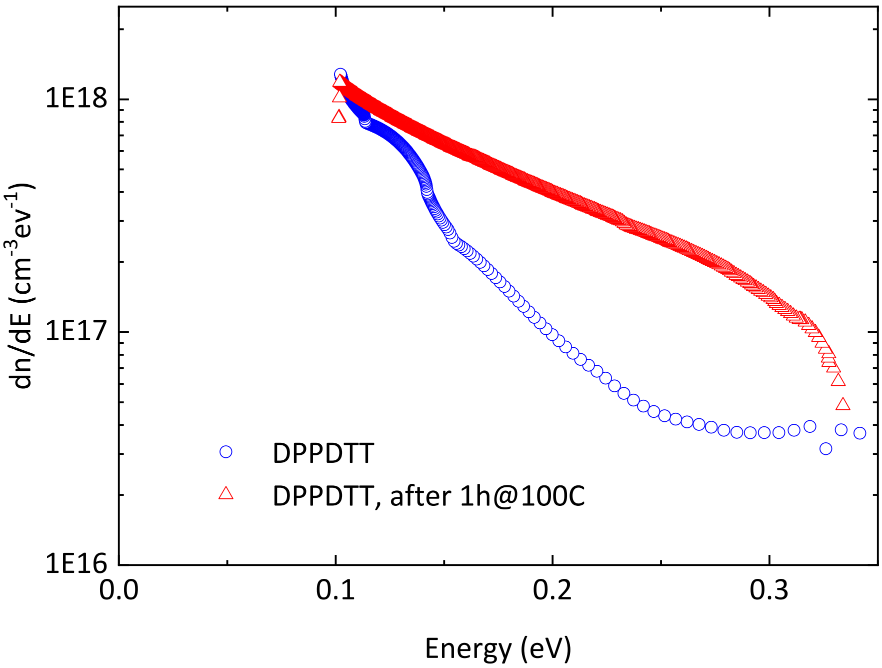

Supplementary Figure 27: Effect of additives on the dn/dE of DPP-DTT. *dn/dE values extracted for a DPP-DTT device with solvent additive (blue circles) and after the solvent additive has been removed (red triangles).*

Supplementary Note 7 – TD-SCLC measurements on IDT-BT

We measured IDT-BT SCLC diodes (thickness 110 nm) fabricated with a solvent additive in the temperature range from 300K to 160K. The extracted activation energy as well as computed E_D values for a pristine IDT-BT SCLC diode are shown in Supplementary Figure 28.

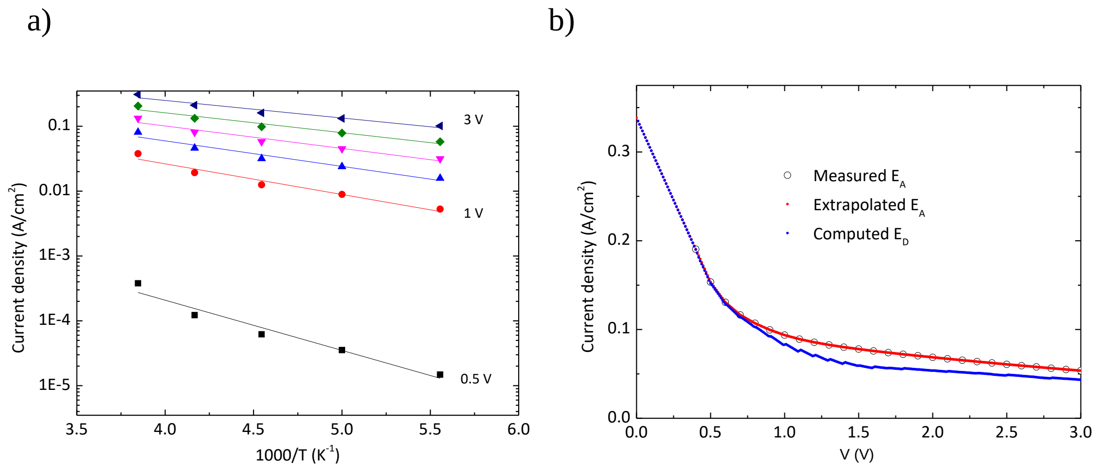

Supplementary Figure 28: Activation energy of IDT-BT diodes with additive. *a) Arrhenius-type plot of the temperature dependence of the current density at different applied voltages for a pristine IDT-BT diode with a solvent additive; b) Corresponding extracted activation energy* E_A *as well as computed* E_D *values.*

We subsequently stored our devices in vacuum for 3d to remove the solvent additive completely. The extracted activation energy as well as computed E_D values for a IDT-BT SCLC diode with the additive removed are shown in Supplementary Figure 29.

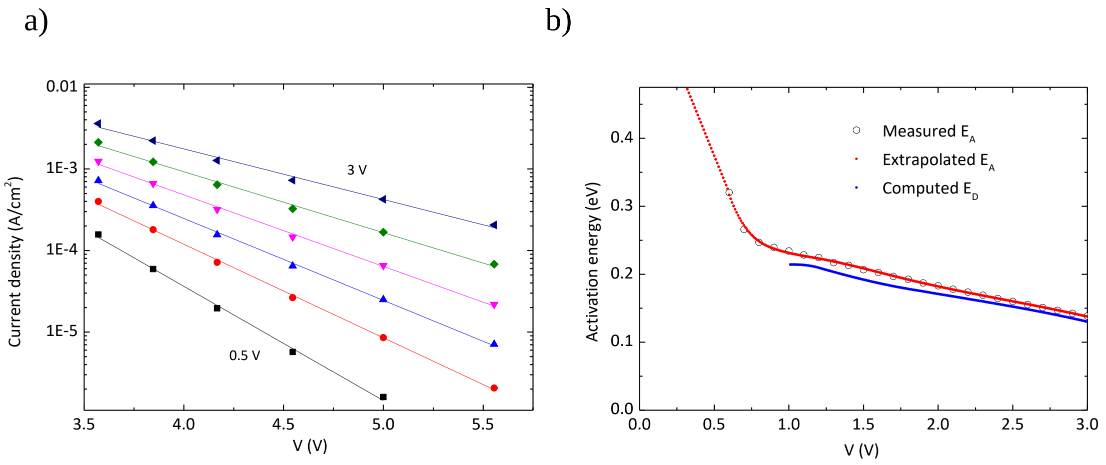

Supplementary Figure 29: Activation energy of IDT-BT diodes without additive. *a) Arrhenius-type plot of the temperature dependence of the current density at different applied voltages for a IDT-BT diode with the solvent additive removed; b) Corresponding extracted activation energy* E_A *as well as computed* E_D *values.*

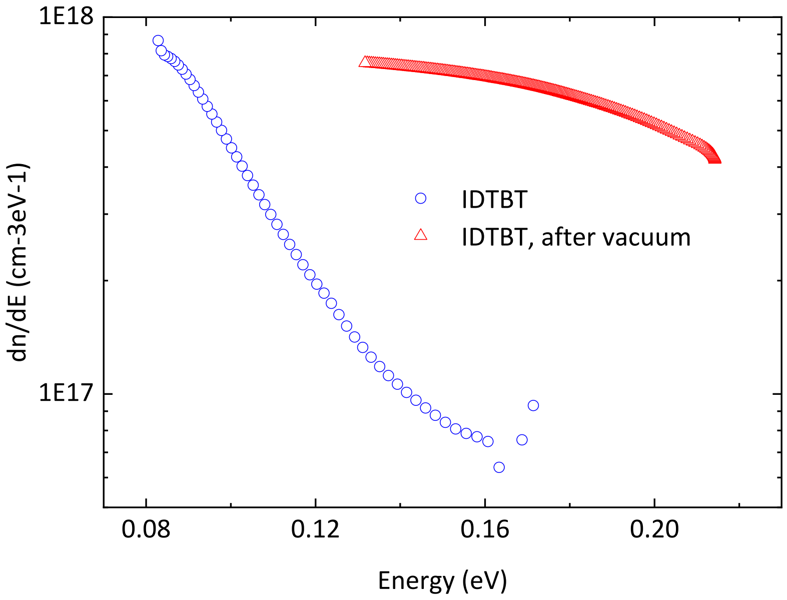

Supplementary Figure 30: Effect of additives on the dn/dE of IDT-BT. *dn/dE values extracted for an IDT-BT device with solvent additive (blue circles) and after the solvent additive has been removed (red triangles).*

Supplementary Note 8 - Microstructure of polymer films

We also performed ellipsometry measurements to determine the film thicknesses independently and to investigate the optical anisotropy due to chains being aligned in the plane of the film.

The film thicknesses extracted for polymer films spun on silicon and gold are shown in Table S1. For DPP-BTz and IDT-BT this gave reliable results consistent with the AFM measurements. For DPP-DTT the film thickness determined by ellipsometry is slightly lower than what we obtained using Atomic Force Microscopy (AFM). This mismatch might also be the explanation why on gold the optical constants for DPP-DTT appear to be isotropic, which is not fully consistent with the GIWAXs data obtained for the polymer (Figure 4).

The thicknesses determined for the different samples are summarized in Table 1.

Sample	DPP-DTT on Si	DPP-DTT on Au	DPP-BTz on Si	DPP-BTz on Au	IDT-BT on Au
Film thickness	82nm	80nm	155nm	170nm	189nm

Supplementary Table 1: Film thickness measured by Ellipsometry. *Film thicknesses of the different samples on Si and on Au*

To determine the uniaxial, anisotropic optical properties the following layered optical model was used: (i) substrate 1mm of Si; (ii) native SiO_2-layer of 1.2nm thickness; (iii) gold of 50nm thickness; (iv) biaxial polymer layer (see Table S1 for thicknesses used).

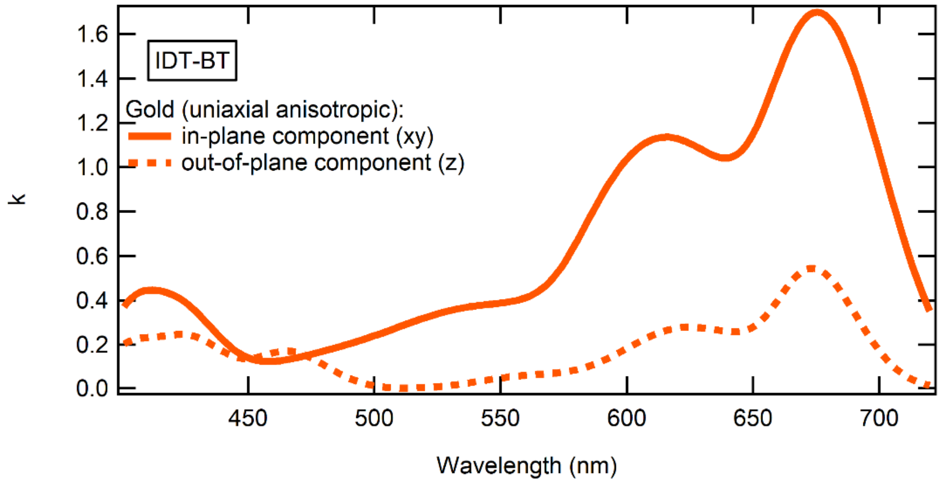

Supplementary Figure 31: Optical constants of IDT-BT. *In-plane (solid line) and out-of-plane (dashed line) component of the imaginary part of the complex refractive index* ***k*** *(extinction coefficient) for IDT-BT on gold.*

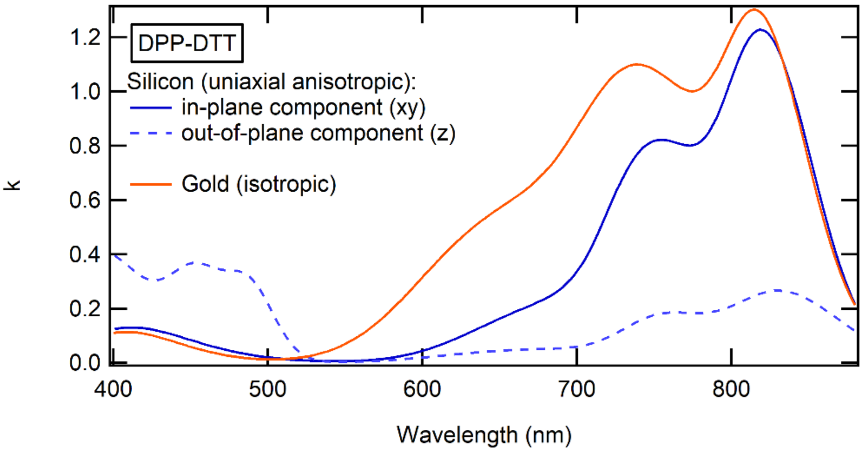

Supplementary Figure 32: Optical constants of DPP-DTT. *Imaginary part* ***k*** *of the complex refractive index of DPP-DTT on Si(100) and on gold. Blue lines: in-plane (blue solid line) and out-of-plane (blue dashed line) component of* ***k*** *of DPP-DTT on Si(100). Orange line:* ***k*** *of DPP-DTT on gold (isotropic).*

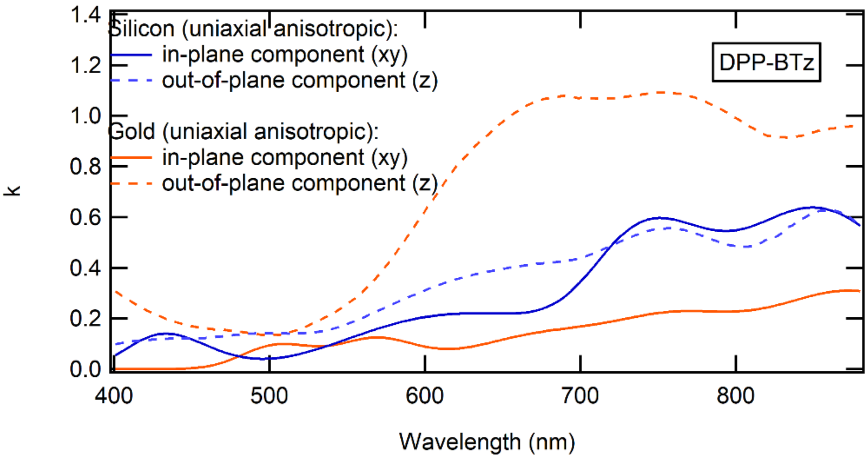

Supplementary Figure 33: Optical constants of DPP-BTz. *Imaginary part* ***k*** *of the complex refractive index of DPP-DTT on Si(100) (blue lines) and on gold (orange lines). Solid lines: in-plane component. Dashed lines: out-of-plane component.*

For films on SiO_2 substrates, the spectra shown in Supplementary Figures 31-33 show clearly that, as expected and consistent with the GIWAXS measurements, the polymer chains are preferentially oriented in the plane of the film. However, the ellipsometry data on gold substrates for DPP-DTT and DPP-BTz would suggest a more isotropic orientation or even out-of-plane chain orientation (in the case of DPP-BTz) on gold substrates. This is inconsistent with the GIWAXS measurements and is likely to be an artefact due to shortcomings of the complex multilayer optical model for these semicrystalline polymers that have strong edge-on and face-on orientation. For this reason we relied for the microstructure characterization mainly on the GIWAXS measurements presented in Fig. 4 and we used the ellipsometry measurements mainly to cross-check film thickness values.

Supplementary References

1. Krellner, C. *et al.* Density of bulk trap states in organic semiconductor crystals: Discrete levels induced by oxygen in rubrene. *Phys. Rev. B - Condens. Matter Mater. Phys.* **75,** 1–5 (2007).
2. Schauer, F., Novotny, R. & Nešpůrek, S. Space-charge-limited-current spectroscopy: Possibilities and limitations. *J. Appl. Phys.* **81,** 1244–1249 (1997).
3. Schauer, F., Nešpůrek, S. & Valerián, H. Temperature dependent space-charge-limited currents in amorphous and disordered semiconductors. *J. Appl. Phys.* **80,** 880–888 (1996).
4. Hoerl, A. E. & Kennard, R. W. Ridge Regression: Biased Estimation for Nonorthogonal Problems. *Technometrics* **12,** 55–67 (1970).
5. Deutsch, M. & Beniaminy, I. High accuracy deconvolution method using spline functions. *Rev. Sci. Instrum.* **53,** 90–97 (1982).
6. Katsouras, I. *et al.* Charge transport in poly(p-phenylene vinylene) at low temperature and high electric field. *Org. Electron.* **14,** 1591–1596 (2013).
7. Abbaszadeh, D. *et al.* Elimination of charge carrier trapping in diluted semiconductors. *Nat. Mater.* **15,** 628–633 (2016).
